# Reconstruction of Scaphoid Nonunion Fractures of the Proximal One Third With a Vascularized Bone Graft From the Distal Radius

**Published:** 2014-06-27

**Authors:** Frank Werdin, Patrick Jaminet, Beate Naegele, Matthias Pfau, Hans-Eberhard Schaller

**Affiliations:** Department for Hand, Plastic, Reconstructive Surgery, Burn Center, BG-Trauma Center, University of Tübingen, Tübingen, Germany

**Keywords:** scaphoid, nonunion, bone graft, vascularized, reconstruction

## Abstract

**Objective:** The treatment of proximal located scaphoid nonunion is a well-known and common problem. For these patients, we used a vascular pedicled bone graft of the distal radius. **Methods:** In the last 7 years, 75 patients were treated with the vascular pedicled bone graft. Retrospectively, patients’ data, healing rates, and factors influencing scaphoid healing were analyzed. **Results:** The overall healing rate in cases with proximal located nonunions (n = 54) was approximately 70%. Out of these 54 patients, 47 patients showed avascular proximal fragments. Multivariate analysis showed no significant impact for the factors age, smoking, duration of disease, or previous operation. **Conclusions:** In our negative selected patient group, we were able to achieve good results with the usage of a pedicled vascularized bone graft of the distal radius. Our results indicate a favorable outcome for the use of a pedicled vascularized distal radius bone graft in both scaphoid nonunion fractures of the proximal third, with or without an avascular proximal pole.

Scaphoid nonunion is a well known and common problem in trauma and hand surgery.^1^^,^^2^ In the last years, the introduction of improved instrumentation and operative techniques has led to increased healing rates.^3^^,^^4^ However, the treatment of scaphoid nonunion of the proximal pole with a sclerotic or avascular fragment remains a persisting problem. Optimal stabilization and vascularization is necessary to achieve good operative outcomes. It is well known that the vascularity of the proximal pole depends on sufficient intraosseous blood supply.^5^ This is likely to be compromised following fracture, presenting the main reason for the high incidence of scaphoid nonunion and avascular necrosis of the proximal pole. Nonunion occurs in 10% to 15% of patients if a scaphoid fracture is not detected and treated. Nonunion of proximal pole fractures can range of up to 30%.^3^^,^^5^

Reported healing rates after surgical reconstruction vary from 80% to 100% for middle and distal third nonunion fractures, with only a union rate of 64% to 77% for proximal third nonunions.^2^^,^^6^^,^^7^ Irrelevant of the location of the nonunion, vascularization, duration of disease, and operative methods remain important factors influencing healing rates.^8^ To improve the outcome after surgical treatment of proximal scaphoid nonunion, different operative methods have been described, from the Matti-Russe's plasty to the use of a free microvascular bone graft,^8^ which represents the most extensive method.

Less complex—and quite elegant—is the usage of a pedicled vascularized bone graft of the distal radius to revascularize the proximal pole.^1^ Basic anatomic knowledge of the forearm and wrist blood supply is well known since the 19th century. The importance of the A. carpi transversa palmare for surgical procedures of the wrist was first described in 1987 by Kuhlman.^9^ Further anatomical studies and first clinical implementation of a pedicled vascularized bone graft from the palmar distal radius were performed by Mathoulin and Haerle.^10^ Zaidemberg was first to describe a dorsal pedicled bone graft based on the 1,2 intercompartmental supraretinacular artery.^11^

In our department for hand, plastic, and reconstructive surgery, we developed an algorithm for the treatment of scaphoid nonunion.^3^ On the basis of this algorithm, we use the vascularized pedicled bone graft from the distal radius in cases with avascular sclerotic or very little proximal pole fragments and in cases of recurrent scaphoid nonunion.

During 7 years, we treated 75 patients with scaphoid nonunion, using either dorsal or palmar pedicled bone grafts from the distal radius. The aim of this retrospective study was to evaluate the overall healing rates of scaphoid nonunion fractures of the proximal one third. Furthermore, we analyzed factors influencing the healing rates and redefined indications for this operative procedure.

## METHODS AND PATIENTS

Over a period of 7 years, 323 patients with scaphoid nonunion fractures were treated in the BG-Trauma Center, University of Tübingen.

In a retrospective study, patient data were analyzed regarding age and sex, medical history (previous operations of the scaphoid), operative technique (palmar/dorsal approach, Herbert screw/k-wire), time of immobilization, range of motion (preoperative and 12 weeks postoperative), and healing rates. Healing of the scaphoid was determined by x-ray examination after 3 months. In cases with unclear diagnostic findings in x-ray investigation (not in all projections trabeculae across the nonunion site), computed tomographic (CT) scan or magnetic resonance imaging (MRI) was performed. Patients with scaphoid lunate advanced collapse (SLAC) wrist or advanced wrist arthritis were excluded from this study.

Healing rates were analyzed and presented by means and percentages. Significance of data between 2 groups was evaluated using the Wilcoxon test, Fisher exact test, and regression analysis. Significance was determined with *P* < .1. For the evaluation of factors influencing healing rates, multivariate analysis was performed. The following factors were analyzed: age, smoking, duration of disease, operative treatment (screw/wire fixation), first operative/previous operative intervention.

Statistical analysis was performed in cooperation with Dr biol hum Christoph Meisner, Institut für Medizinische Biometrie der Eberhard-Karls-Universität Tübingen.

### Anatomy and operative protocol

**Figure 1:**
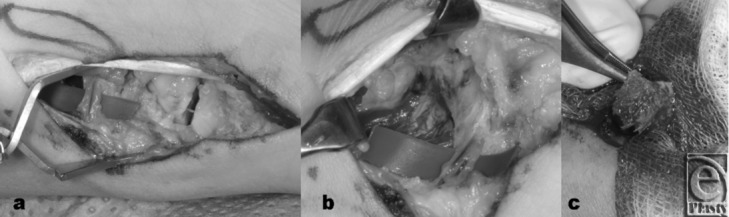
Intraoperative situs. A 37-year-old male patient suffering from nonunion for 3.5 years. (*a*) Overview with the scaphoid nonunion right and the green marked vascular pedicle left. (*b*) Detail out of *a*; vascular pedicle at the border of the musculus pronator quadratus. (*c*) Detailed picture after harvesting of the vascular bone graft.

In our department, the palmar approach was selected as first choice. The palmar carpal arch (Arteria carpi transversa) arises in the distal forearm at a median of 1.5-cm proximal the tip of the radial styloid. This artery, with its venae comitantes, crosses the palmar aspect of the distal forearm, close to the radiocarpal joint and just distal to the pronator quadratus muscle (Figs 1a and 1b). It runs inside a periosteal-capsular membrane of the radioulnar joint. At this level, a “T-shaped” anastomosis with the anterior branch of the anterior interosseous artery is always found. The palmar carpal arch runs ulnarly until it reaches the ulnar artery. However, the radial part is usually dominant. The diameter varies between 0.5 and 1.0 mm. The palmar carpal arch gives origin, especially in its ulnar part, to numerous periosteal and cortical perforators.^9^

If the origin of the radial part of the palmar carpal arch, as in most cases, is just proximal to the radiocarpal joint, the bone graft is harvested on the palmar-ulnar aspect of the radius, close to the distal radioulnar joint. The pivot point of the pedicle is its origin from the radial artery. The length of the pedicle is sufficient enough to allow exact placement of the bone graft into the scaphoid without flexion of the wrist (Fig 1). In cases with previous palmar operative access or very small proximal scaphoid fragments, the dorsal approach is preferred. The pedicled bone graft is harvested in a similar manner, based either on the 1,2 intercompartmental supraretinacular artery or on an artery on the bottom of the third or fourth extensor loge.

**Figure 2:**
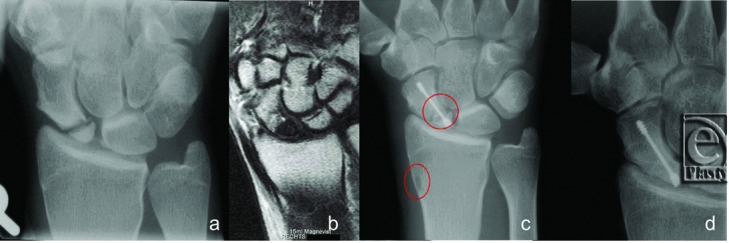
X-rays of a proximal located scaphoid nonunion. A 25-year-old male patient suffering from scaphoid nonunion for 1.5 years. (*a*) Preoperatively. (*b*) Preoperative MRI with avascular proximal fragment. (c) Intraoperative aspect with the mini-Herbert screw after dorsal incision, red marked is the harvesting location of the bone graft and its new location in the nonunion site. (d) X-ray investigation after 12 weeks.

**Figure 3:**
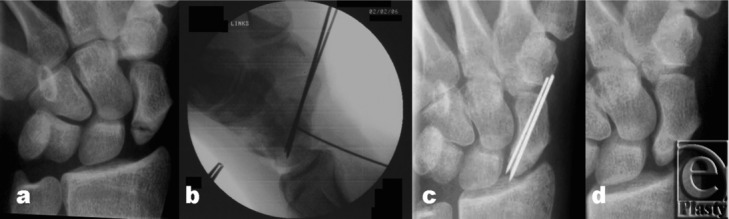
X-rays of a proximal located scaphoid nonunion. A 28-year-old male patient suffering from scaphoid nonunion for 2 years. (*a*) Preoperatively. (*b*) Intraoperatively with a small k-wire for fixation of the bone graft, palmar approach. (*c*) 6 weeks postoperatively. (*d*) 12 weeks postoperatively.

In both methods, additional internal stabilization of the scaphoid was performed, either by using a Herbert screw (first choice method) (Fig 2) or with Kirschner-wire transfixation (Fig 3). After resection of the nonunion zone, the vascularized bone was fixed in its new position either by simple compression of Herbert screw or by an additional 0.6-mm wire (Fig 3a).

### Clinical practice

The vascularized bone graft from the distal radius has been used either in patients with scaphoid nonunions of the proximal pole with little or sclerotic fragments or in patients with scaphoid nonunion recurrence after previous operative therapy. Preoperatively all patients underwent x-ray investigations (posterior/anterior and lateral projection of the wrist as well as 4 projections of the scaphoid). If necessary (eg, avascular necrosis of the proximal fragment or hump back deformity), additional CT scan or MRI (contrast agent) for planning of the operation was performed. Patients with any advanced form of Scaphoid nonunion advanced collaps (SNAC) wrist did not undergo reconstruction of the scaphoid at all.

After the operation, all patients underwent x-ray follow-ups at 6, 12, and optionally 24 weeks postoperatively. In general, the minimal time of immobilization was 6 weeks, varying up to 12 weeks, depending on the results of x-ray investigation. Scaphoid healing was determined if trabeculae across the nonunion site were present on all projections (posterior/anterior and lateral projection of the wrist as well as 4 projections of the scaphoid). When scaphoid healing could not be definitely confirmed or excluded by x-rays (definite healing: trabeculae across the nonunion site in all projections; possible healing: no gap visible; no healing: gap visible), a subsequent CT scan or MRI was performed after 12 weeks.

### Patients

In the last 7 years, 323 patients underwent operative reconstruction of scaphoid nonunion fractures. Seventy-five (22%) patients received scaphoid reconstruction with a vascularized bone graft from the distal radius. Five patients were lost in follow-up. Indications for scaphoid reconstruction were nonunions of the distal (n = 2), the middle (n = 14), and proximal (n = 54) third. Healing rates of the distal and middle located scaphoid nonunions were varying between 92% (middle) and 100% (distal). This study focused on proximally located nonunion fractures. Accordingly, 54 patients were included in this study.

The average age of the 54 patients was 29.7 years (17-57); 50 of them (92.6%) were male and 4 (7.4%) were female. The average time between trauma and final reconstruction of the scaphoid nonunion (duration of disease) was 39 (6-166) months.

Nine patients (16.7%) suffered from persistent scaphoid nonunion after previous surgery. Out of these 9 patients with persistence, 5 patients received previous Matty Russe's plasty and 4 patients received Herbert screw osteosynthesis.

The scaphoid reconstruction was performed in 45 cases (83.3%) using a palmar and in 9 (16.7%) cases using a dorsal approach. These latter cases presented with a very small proximal fragment. Of 54 patients, 47 patients suffered from avascular, small, and sclerotic proximal pole fragments. Avascular pole fragments were confirmed either by preoperatively MRI (n = 12) or intraoperatively (small and sclerotic fragments with absence of bleeding out of the proximal bone after opening of the Esmarch ischaemia).

Herbert screw (in 18 cases) and k-wire (in 36 cases) fixation were used for internal stabilization of the scaphoid. The average operating time was 128 minutes (85-140 minutes), the average duration of immobilization was 11.8 weeks (6-17). K-wires were removed after 12 weeks in all 36 patients.

## RESULTS

The overall union rate after 3 months was 68.5% (37 patients). The union rate in the group of avascular fragments (n = 47) was 71%. In 45 cases, definite scaphoid healing or nonhealing was confirmed by x-rays. In 17 cases, additional CT scan of the wrist and in 2 cases additional MRI of the wrist were performed to rule out or confirm persisting scaphoid nonunion.

The average duration of disease in patients with healed scaphoid nonunion was 1.7 years and in patients with persisting scaphoid nonunions (nonhealing) 3.4 years. However, their multivariate analysis showed no significant influence for the factor “duration of diseases.”

Forty patients were younger than 30 years and had a healing rate of 72%. Fourteen patients were older than 30 years and had a healing rate of 64.2%. Univariate analysis showed no significant difference (*P* > .1) between these 2 groups. Multivariate analysis for the factor “patient age” was negative (*P* = .133).

Interestingly, there were no significant differences (*P* = .773) between the healing rates in patients without previous treatment for scaphoid nonunion compared with patients treated operatively before (recurrence of scaphoid nonunion).

According to the fixation method (osteosynthesis), the healing rates were consecutively 66.7% for the Herbert screw and 69.4% for the K-wire transfixation (no significant difference; *P* > .1). The multivariate analysis for the factors age (*P* > .1) and smoking (*P* > .1) was also negative.

## DISCUSSION

Over the last years, different methods for scaphoid nonunion reconstruction have been described. Because of the frequency of the disease, a successful method for daily operative practice is necessary. In our department, internal fixation with Herbert screw, in addition to a nonvascularized bone graft from the iliac crest, has become the criterion standard in surgical treatment of scaphoid nonunion. In accordance with other authors, we can report high healing rates with this standardized method of up to 88%.^3^

Until now, according to our algorithms for the reconstruction of scaphoid nonunion,^3^ there are 2 indications for the additional use of a pedicled vascularized bone graft from the distal part of the radius:

1. The presence of a small or avascular proximal pole fragment
2. Recurrence of scaphoid nonunion after previous surgery

The study of recent literature showed healing rates between 47% and 100% for scaphoid nonunion surgical treatment, using nonvascularized bone grafts and screw fixation.^12^ Important factors for bone healing are the vascularity of the proximal pole as well as the stage of the nonunion.^13^ Green demonstrated a healing rate of 92% when the proximal pole showed intraoperative bleeding and a rate of subsequent nonunion of 100% when the proximal pole was avascular.^14^ Despite the chance that an ischemic proximal pole is prone to revascularization using the Russe inlay or anterior wedge graft, these methods are unpredictable.

The possibility of proximal pole revascularization and a quicker union timeframe had been shown when using pedicled vascular bone grafts from the distal radius.^1^^,^^15^^,^^16^ Two factors are known to improve bone healing conditions: better local blood flow and supply of cells.

Several pedicled grafts have been described. A distal radial graft based on the 1,2 intercompartmental supraretinacular artery pedicle showed a healing rate of 6 out of 10 patients.^17^ Straw et al could only demonstrate a healing rate of 27% using the same technique.^18^ Excellent results have been achieved when using a vascularized graft from the palmar and ulnar aspect of the distal part of the radius, supplied by the palmar carpal artery. Malizos et al could demonstrate an overall healing rate of 100% using either dorsal or volar grafts in 30 patients.^19^ Despite these favorable results, vascular bone grafts are not indicated in SLAC wrists or advanced wrist arthritis.^20^ According to the literature, other factors influencing healing rates of scaphoid nonunions are duration of disease, previous operations, patients age, and smoking.^5^^,^^12^^,^^21^

In our department, 75 of 323 patients underwent scaphoid reconstruction using a vascularized bone graft from the distal radius throughout the last 7 years. It seems to be the largest series ever reported.

Fifty-four patients suffered from a very proximal location of the nonunion. Out of these, 47 patients had an avascular proximal fragment. According to other authors,^12^^,^^14^^,^^21^^,^^22^ cases with little proximal fragments, long time between accident and operation, and recurrence of nonunion after previous operations present a persisting problem in the operative treatment of scaphoid nonunion, leading to poor healing rates. By using a vascularized pedicled bone graft from the distal radius, we can report a healing rate of almost 70% in this negative selected collective.

Comparing our results with our own healing rates in cases of distal or middle located nonunions, proximal location seems to be an important negative predictor concerning the healing rates. This matches favorable to the results of Inoue et al^23^ or Ramamurthy et al.^22^ Therefore, in accordance to literature,^12^^,^^13^^,^^21^^,^^24^ we had evaluated the location of scaphoid nonunion furthermore as a prognostic factor concerning scaphoid healing. In our group of avascular fragments (n = 47), we were able to achieve a healing rate of 71% (n = 33). On the basis of our clinical practice and the retrospective design of the study, we were not able to confirm revascularization of the avascular pole fragment in all 33 patients by MRI. Two patients with MRI showed sufficient vascularization of the proximal pole. However, in our opinion, healing, even if it is simply confirmed by x-rays, is only possible in cases of revascularized proximal pole fragments. These results can be likened to those of Sauerbier et al.^1^ They reported 70% healing rates in cases with avascular fragments using a pedicled bone graft from the distal radius.^1^

Unfortunately, it was not possible to compare the healing rates in relation to the operative approach (dorsal/palmar). On one hand, the group of patients with the dorsal approach was too small, on the other hand, all patients with the dorsal approach presented with small avascular proximal fragments (negative predictor).

Surprisingly, there was no significant difference in healing between Kirschner wire stabilization and the use of Herbert (compression) screw. We initially expected better healing rates in cases with screw osteosynthesis, due to the high compression and consecutive better stabilization of the scaphoid.

In contrast to literature,^12^^,^^22^^,^^23^^,^^24^ multivariate analysis did not reveal any significant impact for the factors “duration of disease” and “previous operation” as well as “smoking” and “age” on healing rates. However, out of 45 patients without previous operation, 38 patients suffered from an avascular nonunion of the proximal one third. Also, all 11 patients with a short period between trauma and operative treatment and persisting nonunion suffered from a proximal location nonunion, with avascular proximal fragments. Considering literature and our general experience (323 operations in patients with scaphoid nonunion), we still value both factors as negative predictors. However, our results underline the strong impact of location and vascularity on healing rates of fractures of the scaphoid.

Finally, we compared our results with other methods—for example, using free vascularized bone grafts from the distal part of the femur or the iliac crest. These studies report healing rates between 80% and 100%.^25^^-^28 However, one must keep in mind that these results are based on examination of a small number of patients (eg, n = 12). No difference in union rates was described between the use of free vascularized grafts from the femur compared with grafts from the iliac crest. Jones reported of a healing rate of 100% in 12 patients treated with a bone graft from the distal femur condylus.^28^ In another study, Jones et al compared these healing rates with those of 10 patients with a pedicled radius bone graft. He described a significant positive impact on the healing rates and healing time in patients with the free vascular bone graft. He reported that 4 of 10 patients achieved healing by using the pedicled bone graft of the distal radius.^29^

Our own experience with the free vascular femur condylus showed a healing rate of 75%. Actually we have chosen this method for patients with previous operation and avascular pole fragments. However, we also know about the disadvantages of this technical challenging operation. We could notice a longer operation time, higher morbidity of the donor side, and a considerable limitation in the range of motion postoperatively in these patients. Therefore, we prefer the pedicled bone graft of the distal part of the radius as the method of first choice for the aforementioned difficult cases.

Limitation of our study is the retrospective design and the absence of CT scan or MRI in all patients. However, the aim of this clinical control study was to evaluate our results concerning healing rates and to redefine our indications for this operative procedure. In our opinion, limitations are compensated by the size of our study presenting a very large series of scaphoid reconstruction with a pedicled bone graft from the distal radius. Therefore, we think we achieved our initial aims and our results are comparable with those of other studies.

Our study shows that a high rate of union can be reached with the pedicled bone graft, even in the presence of a negative selected patient group. Our study supports the indication for the use of a vascularized bone graft from the distal part of the radius in proximal nonunion fractures of the scaphoid, with or without avascular proximal fragments, especially in combination with previous unsuccessful operations. Intraoperatively, Herbert screw and k-wire osteosynthesis performed equally well. In cases of persisting nonunion after pedicled bone graft, free vascular bone grafts could be an alternative for some of these patients.
